# Supplementary material for: Prenatal Choline Supplementation Alters One Carbon Metabolites in a Rat Model of Periconceptional Alcohol Exposure
Source: Nutrients. 2022 Apr 29;14(9):1874. doi: 10.3390/nu14091874 (PMC9100923; doi:10.3390/nu14091874)
Supplement: Supplementary file 1 [file nutrients-14-01874-s001.zip › nutrients-1686368-supplementary.pdf]

Table S1: Primers used for real-time quantitative PCR analysis of gene expression.

**Gene name**	**Gene Symbol**	**Primer pair ID**	**Accession number**
β – actin[a]	*Actb*	R_Actb_1	NM_031144
ribosomal protein L19[a]	*Rpl19*	R_Rpl19_1	NM_031103
5-methyltetrahydrofolate-homocysteine methyltransferase	*Mtr*	R_Mtr_1	NM_030864
5-methyltetrahydrofolate-homocysteine methyltransferase reductase	*Mtrr*	R_Mtrr_1	NM_001039003
methylene tetrahydrofolate reductase	*Mthfr*	R_Mthfr_1	XM_001074061
betaine-homocysteine methyltransferase	*Bhmt*	R_Bhmt_1	NM_030850
phosphatidylethanolamine N-methyltransferase	*Pemt*	R_Pemt_1	NM_013003

All primers were KiCqStart SYBR Green primer pairs from Sigma-Aldrich, Saint Louis, MO, USA.

[a] Endogenous control.

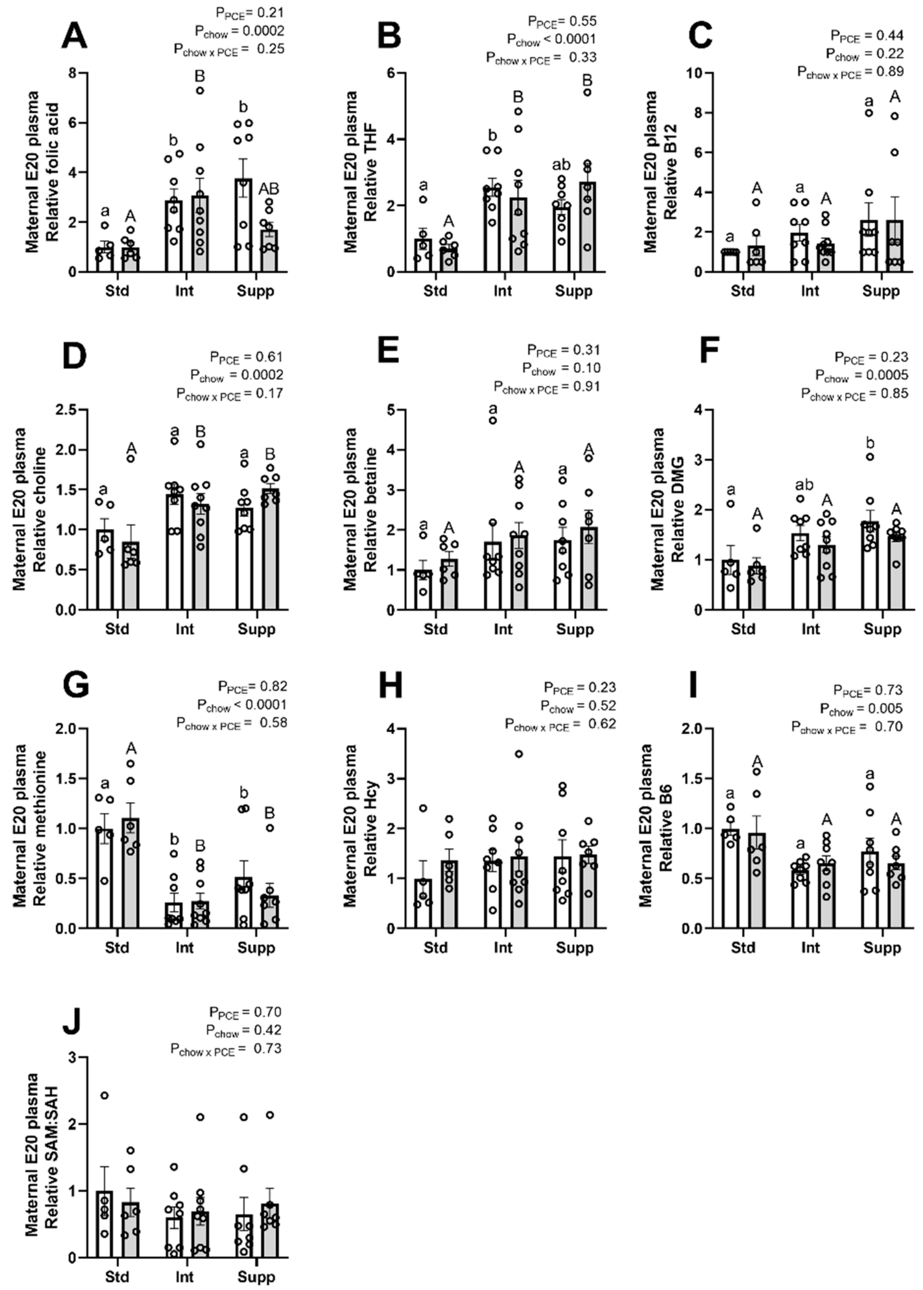

Figure S1: The effects of periconceptional ethanol and choline supplementation on specific components of the folate cycle (A-C), choline pathway (D-F), and methionine cycle (G-J) in late gestation maternal plasma. A) folic acid; B) tetrahydrofolate (THF); C) vitamin B12; D) choline; E) betaine; F) dimethylglycine (DMG); G) methionine; H) homocysteine (Hcy); I) vitamin B6; J) the ratio of s-adenosylmethionine: s-adenosylhomocysteine (SAM:SAH). During the periconceptional period, dams received a control liquid diet (Con; open bars) or a liquid diet containing 12.5% v/v ethanol (PCE; grey bars). For the remainder of pregnancy, dams received either standard chow (Std), intermediate chow (Int) or supplemented chow (Supp), with increasing levels of choline across these groups (1.6 g, 2.6 g, or 7.2 g choline/kg chow), *n* = 5-9 per group. Molecules were measured using mass spectrometry and data expressed as fold-change relative to the average of the Std-Con group (mean ± SEM). Significant differences due to alcohol exposure or chow group were identified by two-way ANOVA and Tukey's post-hoc analysis. Chow effects between Con groups are shown by lower-case letters, and between PCE groups by upper-case letters.

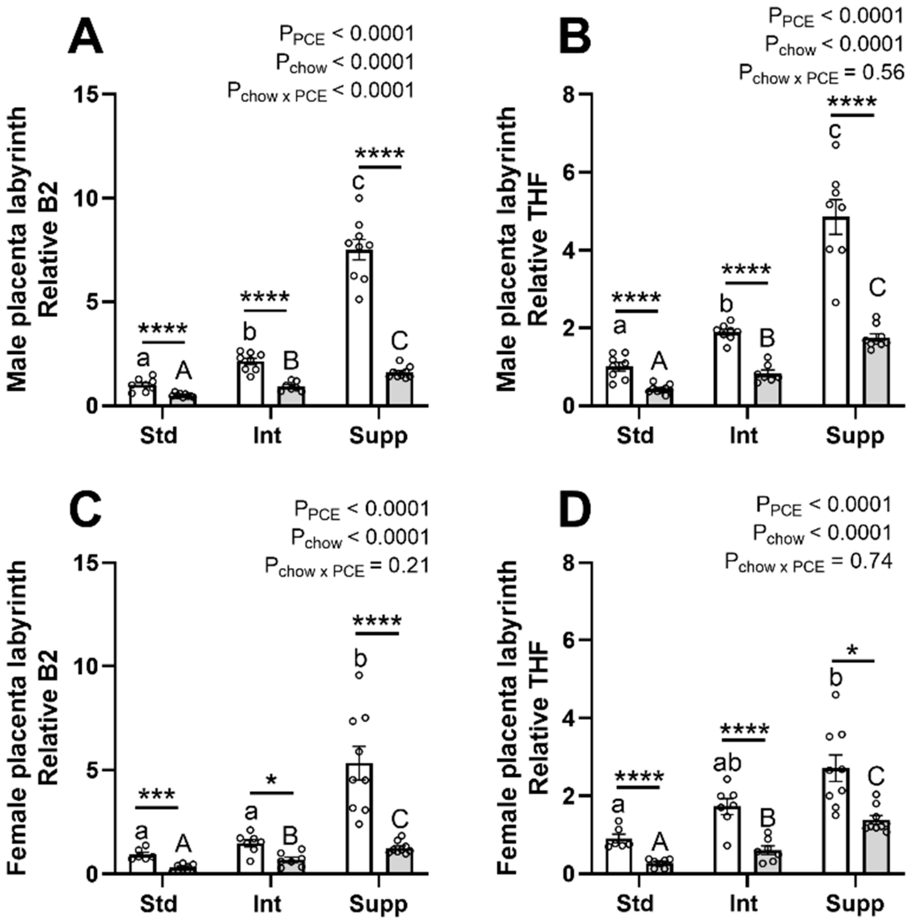

Figure S2: The effects of periconceptional ethanol and maternal choline supplementation on specific components of the folate cycle in placentas of male (A, B) and female (C, D) fetuses. A, C) vitamin B2; B, D) tetrahydrofolate (THF). During the periconceptional period, dams received a control liquid diet (Con; open bars) or a liquid diet containing 12.5% v/v ethanol (PCE; grey bars). For the remainder of pregnancy, dams received either standard chow (Std), intermediate chow (Int) or supplemented chow (Supp), with increasing levels of choline across these groups (1.6 g, 2.6 g, or 7.2 g choline/kg chow), *n* = 6-9 per group. Molecules were measured using mass spectrometry and data expressed as fold-change relative to the average of the male Std-Con group (mean ± SEM). Significant differences due to alcohol exposure or chow group were identified by two-way ANOVA and Tukey's post-hoc analysis. * *P*<0.05; *** *P*<0.001 and **** *P*<0.0001 for differences between Con and PCE within each chow group; chow effects between Con groups are shown by lower-case letters, and between PCE groups by upper-case letters.

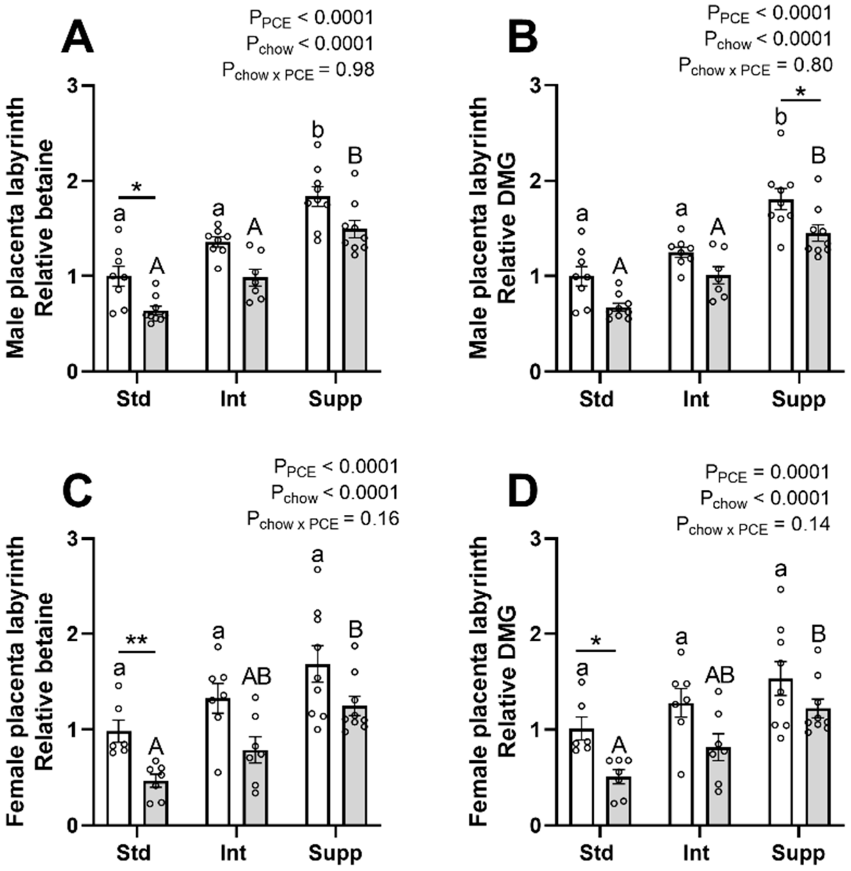

Figure S3: The effects of periconceptional ethanol and maternal choline supplementation on specific components of the choline pathway in placentas of male (A, B) and female (C, D) fetuses. A, C) betaine; B, D) dimethylglycine (DMG). During the periconceptional period, dams received a control liquid diet (Con; open bars) or a liquid diet containing 12.5% v/v ethanol (PCE; grey bars). For the remainder of pregnancy, dams received either standard chow (Std), intermediate chow (Int) or supplemented chow (Supp), with increasing levels of choline across these groups (1.6 g, 2.6 g, or 7.2 g choline/kg chow), n = 6-9 per group. Molecules were measured using mass spectrometry and data expressed as fold-change relative to the average of the male Std-Con group (mean ± SEM). Significant differences due to alcohol exposure or chow group were identified by two-way ANOVA and Tukey's post-hoc analysis. * P<0.05; ** P<0.01 for differences between Con and PCE within each chow group; chow effects between Con groups are shown by lower-case letters, and between PCE groups by upper-case letters.

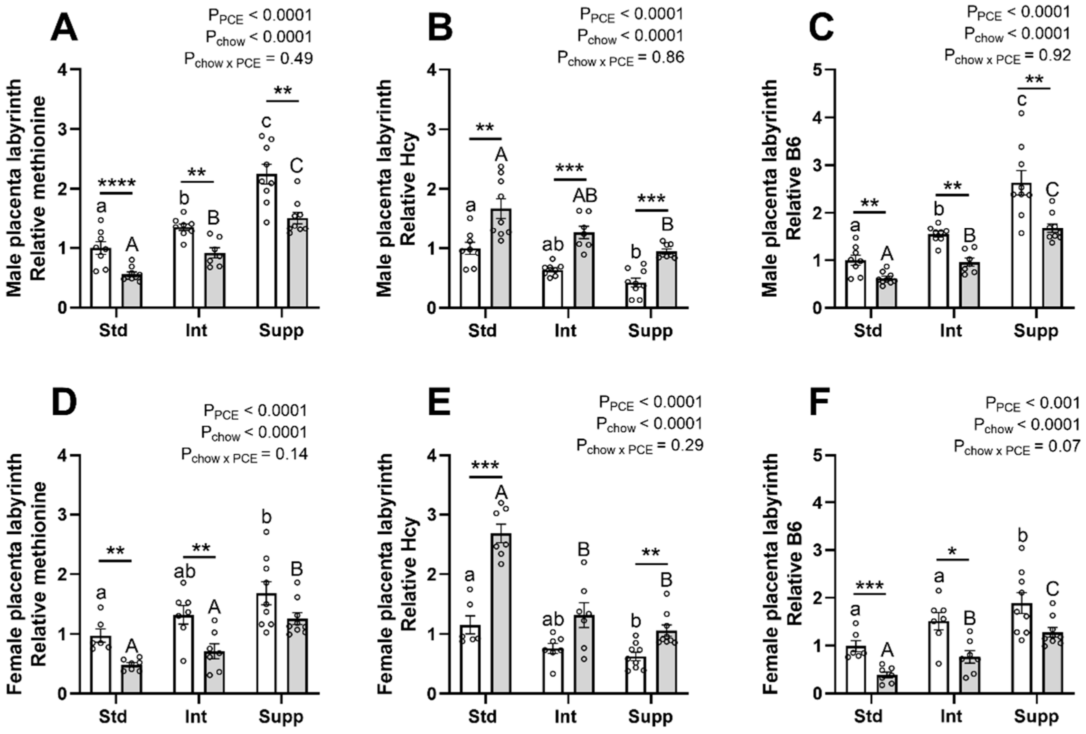

Figure S4: The effects of periconceptional ethanol and maternal choline supplementation on specific components of the methionine cycle in placentas of male (A-C) and female (D-F) fetuses. A, D) methionine; B, E) vitamin B6; C, F) homocysteine (Hcy). During the periconceptional period, dams received a control liquid diet (Con; open bars) or a liquid diet containing 12.5% v/v ethanol (PCE; grey bars). For the remainder of pregnancy, dams received either standard chow (Std), intermediate chow (Int) or supplemented chow (Supp), with increasing levels of choline across these groups (1.6 g, 2.6 g, or 7.2 g choline/kg chow), *n* = 6-9 per group. Molecules were measured using mass spectrometry and data expressed as fold-change relative to the average of the male Std-Con group (mean ± SEM). Significant differences due to alcohol exposure or chow group were identified by two-way ANOVA and Tukey's post-hoc analysis. * P<0.05; ** P<0.01; *** P<0.001 and **** P<0.0001 for differences between Con and PCE within each chow group; chow effects between Con groups are shown by lower-case letters, and between PCE groups by upper-case letters.

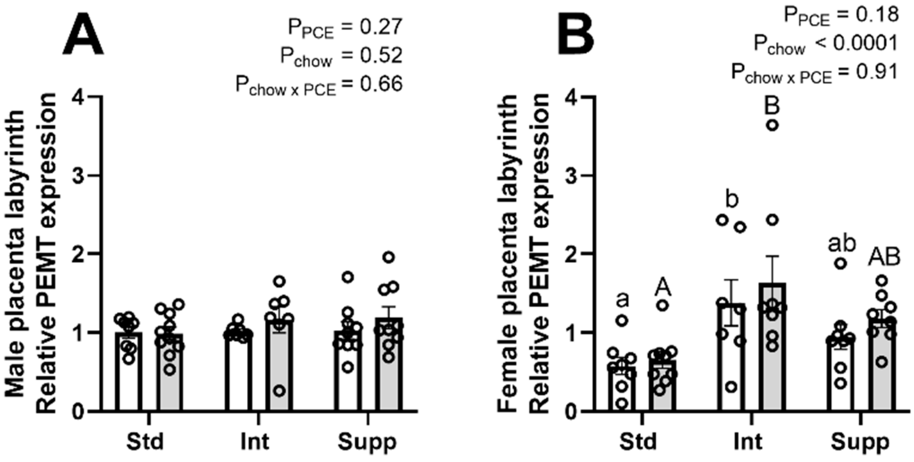

Figure S5: The effects of periconceptional ethanol and maternal choline supplementation on expression of phosphatidylethanolamine methyl transferase (*Pemt*) in placentas of male (A) and female (B) fetuses. During the periconceptional period, dams received a control liquid diet (Con; open bars) or a liquid diet containing 12.5% v/v ethanol (PCE; grey bars). For the remainder of pregnancy, dams received either standard chow (Std), intermediate chow (Int) or supplemented chow (Supp), with increasing levels of choline across these groups (1.6 g, 2.6 g, or 7.2 g choline/kg chow), *n* = 5-9 per group. Gene expression was analysed relative to the geometric mean of *Actb* and *RPL19*, with fold-change relative to the male Std-Con group. All data are presented as mean ± SEM. Significant differences due to alcohol exposure or chow group were identified by two-way ANOVA and Tukey’s post-hoc analysis. Results of post-hoc analysis of chow effects are shown by lower case letters for control groups; upper-case letters for alcohol exposure groups.

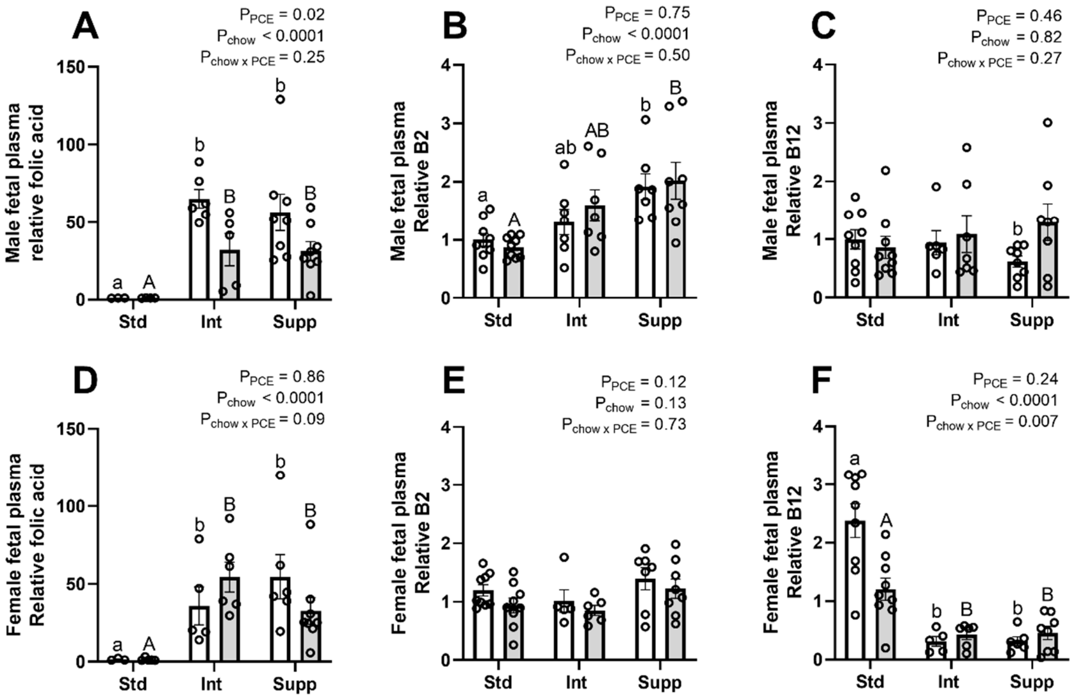

Figure S6: The effects of periconceptional ethanol and maternal choline supplementation on specific components of the folate cycle in male (A-C) and female (D-F) fetal plasma. A, D) folic acid; B, E) vitamin B2; C, F) vitamin B12. During the periconceptional period, dams received a control liquid diet (Con; open bars) or a liquid diet containing 12.5% v/v ethanol (PCE; grey bars). For the remainder of pregnancy, dams received either standard chow (Std), intermediate chow (Int) or supplemented chow (Supp), with increasing levels of choline across these groups (1.6 g, 2.6 g, or 7.2 g choline/kg chow), *n* = 5-9 per group. Molecules were measured using mass spectrometry and data expressed as fold-change relative to the average of the male Std-Con group (mean ± SEM). Significant differences due to alcohol exposure or chow group were identified by two-way ANOVA and Tukey's post-hoc analysis. Chow effects between Con groups are shown by lower-case letters, and between PCE groups by upper-case letters. Peak intensity for folic acid was low, particularly in the Std group plasma and peaks could not be detected in all samples (female Std-Con *n*=3, Std-PCE *n*=5; male Std-Con *n*=3, Std-PCE *n* =4). There were also two missing values in the male Int-PCE group and one in the female Supp-Con group.

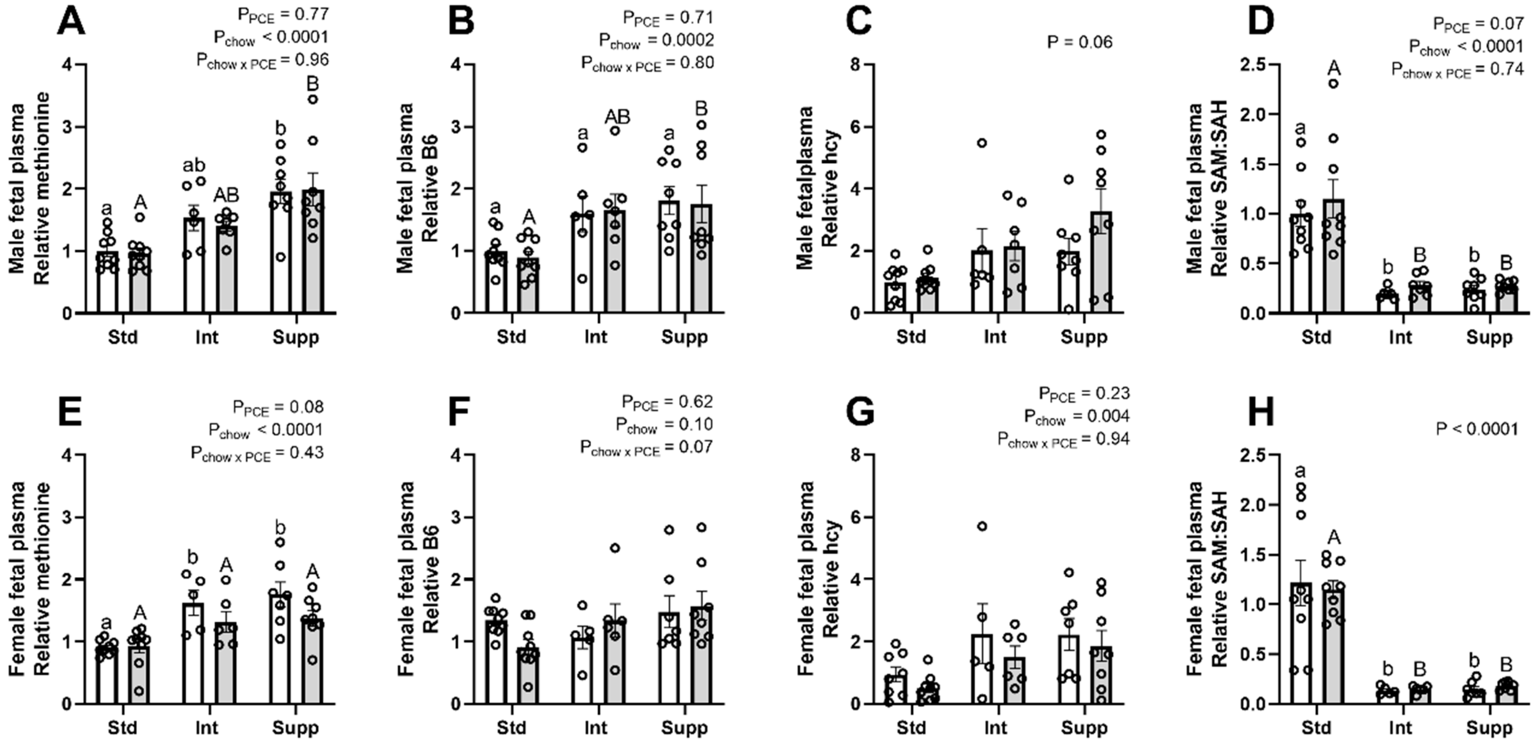

Figure S7: **The effects of periconceptional ethanol and maternal choline supplementation on specific components of the methionine cycle in male (A-D) and female (E-H) fetal plasma.** A, E) methionine; B, F) vitamin B6; C, G) homocysteine (Hcy); D, H) s-adenosylmethionine: s-adenosylhomocysteine ratio (SAM:SAH). During the periconceptional period, dams received a control liquid diet (Con; open bars) or a liquid diet containing 12.5% v/v ethanol (PCE; grey bars). For the remainder of pregnancy, dams received either standard chow (Std), intermediate chow (Int) or supplemented chow (Supp), with increasing levels of choline across these groups (1.6 g, 2.6 g, or 7.2 g choline/kg chow), *n* = 5-9 per group. Molecules were measured using mass spectrometry and data expressed as fold-change relative to the average of the male Std-Con group (mean ± SEM). Significant differences due to alcohol exposure or chow group were identified by two-way ANOVA and Tukey's post-hoc analysis for parametric data, and by Kruskal-Wallis test across all 6 groups, and Dunn's post-hoc analysis for non-parametric data. Results of post-hoc analysis of chow effects are shown by lower case letters for control groups; upper-case letters for alcohol exposure groups.

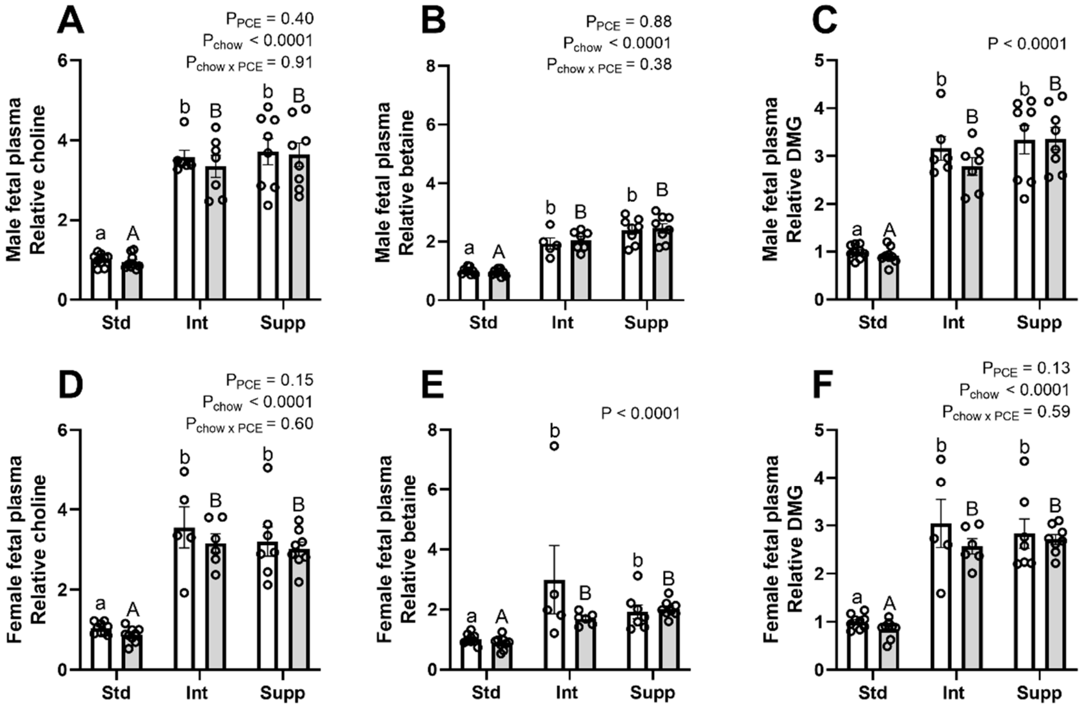

Figure S8: The effects of periconceptional ethanol and maternal choline supplementation on specific components of the choline pathway in male (A-C) and female (D-F) fetal plasma. A, D) choline; B, E) betaine; C, F) dimethylglycine (DMG). During the periconceptional period, dams received a control liquid diet (Con; open bars) or a liquid diet containing 12.5% v/v ethanol (PCE; grey bars). For the remainder of pregnancy, dams received either standard chow (Std), intermediate chow (Int) or supplemented chow (Supp), with increasing levels of choline across these groups (1.6 g, 2.6 g, or 7.2 g choline/kg chow), *n* = 5-9 per group. Molecules were measured using mass spectrometry and data expressed as fold-change relative to the average of the male Std-Con group (mean ± SEM). Significant differences due to alcohol exposure or chow group were identified by two-way ANOVA and Tukey's post-hoc analysis for parametric data; and by Kruskal-Wallis test across all 6 groups, and Dunn's post-hoc analysis for non-parametric data. Results of post-hoc analysis of chow effects are shown by lower case letters for control groups; upper-case letters for alcohol exposure groups.

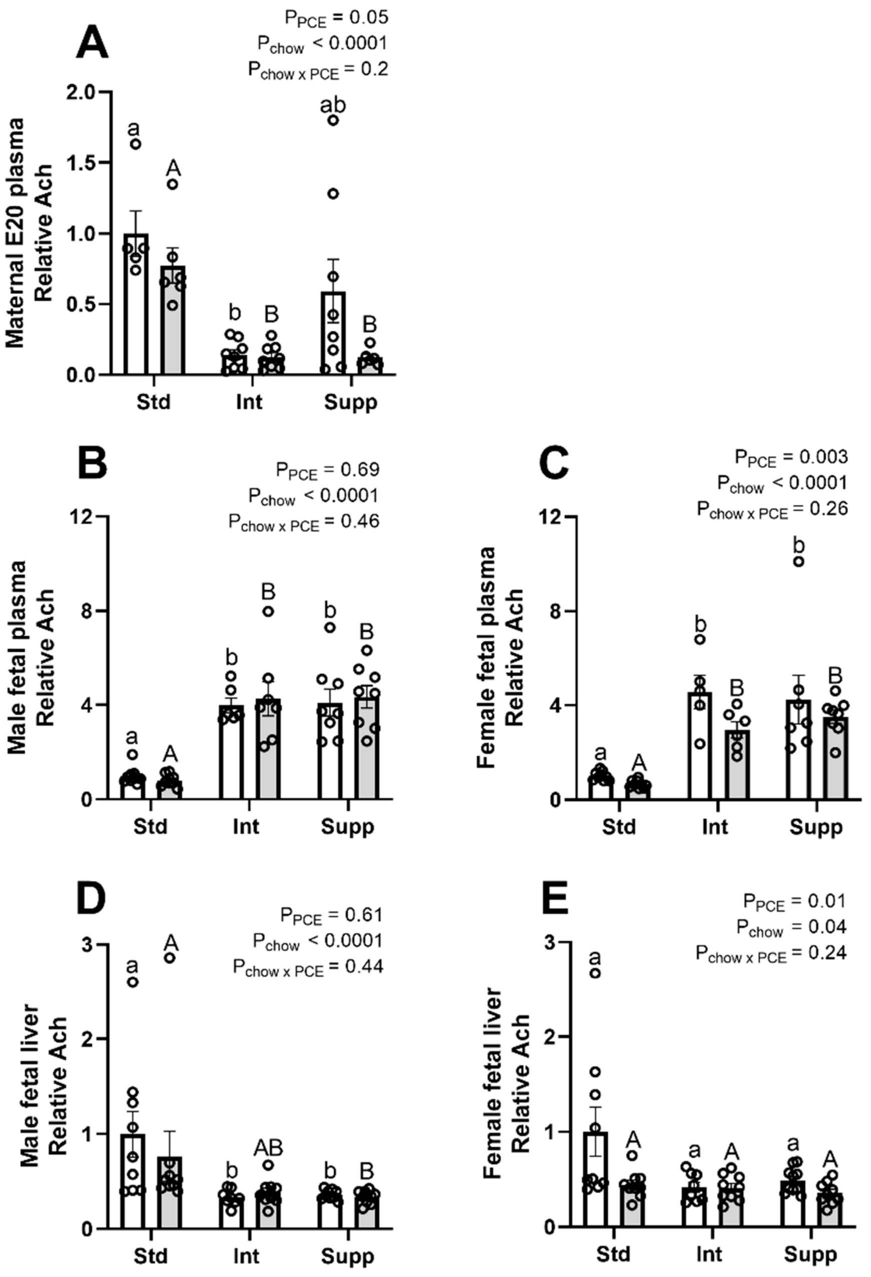

Figure S9: The effects of periconceptional ethanol and choline supplementation on acetylcholine (Ach) levels in maternal plasma (A) fetal plasma (B, D) and fetal liver (C, E). During the periconceptional period, dams received a control liquid diet (Con; open bars) or a liquid diet containing 12.5% v/v ethanol (PCE; grey bars). For the remainder of pregnancy, dams received either standard chow (Std), intermediate chow (Int) or supplemented chow (Supp), with increasing levels of choline across these groups (1.6 g, 2.6 g or 7.2 g choline/kg chow), *n* = 6-9 per group. Molecules were measured using mass spectrometry and data expressed as fold-change relative to the average of the male Std-Con group (mean ± SEM). Significant differences due to chow group were identified by two-way ANOVA and Tukey's post-hoc analysis; chow effects between Con groups are shown by lower-case letters, and between PCE groups by upper-case letters.

Table S2: The effects of periconceptional ethanol and choline supplementation on components of the one-carbon metabolism (1CM) pathway in the fetal liver. During the periconceptional period**,** dams received a control liquid diet (Con) or a liquid diet containing 12.5% v/v ethanol (PCE). Chow levels varied from 1.6 g to 7.2 g choline/kg chow. Molecules measured by mass-spectrometry. Data expressed as fold-change relative to the average of the male Std-Con group (mean ± SEM).

**1CM Molecules**	**Std-Con**	**Std-PCE**	**Int–Con**	**Int-PCE**	**Supp-Con**	**Supp-PCE**	***P Values***
Male	***n = 9***	***n = 9***	***n = 8***	***n = 9***	***n = 9***	***n = 10***	
Vitamin B2	1.0 ± 0.27[a]	0.65 ± 0.27[A]	0.27 ± 0.03[b]	0.24 ± 0.02[B]	0.26 ± 0.02[b]	0.26 ± 0.02[AB]	**P < 0.001[#]**
Vitamin B12	1.0 ± 0.24[a]	0.44 ± 0.11[A]	0.32 ± 0.05[a]	0.32 ± 0.04[A]	0.25 ± 0.05[b]	0.24 ± 0.03[A]	**P < 0.05 [#]**
choline	1.0 ± 0.30[a]	0.56 ± 0.19[A]	0.26 ± 0.02[b]	0.25 ± 0.02[B]	0.25 ± 0.02[b]	0.28 ± 0.02[B]	P_{PCE} = 0.5; **P_{Chow} < 0.001**; $P_{PCE\ x\ chow}$ = 0.2
Betaine	1.0 ± 0.19[a]	0.73 ± 0.13[A]	0.50 ± 0.04[b]	0.50 ± 0.04[B]	0.53 ± 0.04[b]	0.58 ± 0.03[B]	P_{PCE} = 0.8; **P_{Chow} < 0.001**; $P_{PCE\ x\ chow}$ = 0.4
DMG	1.0 ± 0.30[a]	0.57 ± 0.18[A]	0.27 ± 0.02[b]	0.26 ± 0.04[B]	0.28 ± 0.01[b]	0.30 ± 0.02[B]	P_{PCE} = 0.3; **P_{Chow} < 0.001**; $P_{PCE\ x\ chow}$ = 0.3
Methionine	1.0 ± 0.24[a]	0.60 ± 0.09[A]	0.47 ± 0.05[a]	0.40 ± 0.04[A]	0.45 ± 0.03[a]	0.45 ± 0.04[A]	P_{PCE} = 0.2; **P_{Chow} < 0.05**; $P_{PCE\ x\ chow}$ = 0.6
Hcy	1.0 ± 0.22[a]	0.80 ± 0.27[A]	1.48 ± 0.25[a]	1.16 ± 0.26[AB]	1.28 ± 0.13[a]	1.95 ± 0.27[B]	P_{PCE} = 0.8; **P_{Chow} < 0.05**; $P_{PCE\ x\ chow}$ = 0.09
Vitamin B6	1.0 ± 0.22[a]	0.20 ± 0.04[A]	0.26 ± 0.04[b]	0.37 ± 0.05[A]	0.21 ± 0.03[b]	0.32 ± 0.06[A]	**P < 0.05 [#]**
SAM:SAH [^]	1.0 ± 0.22	1.25 ± 0.23	2.27 ± 0.60	1.45 ± 0.42	1.43 ± 0.40	1.40 ± 0.42	P_{PCE} = 0.4; P_{Chow} = 0.5; $P_{PCE\ x\ chow}$ = 0.5
Female	***n = 9***	***n = 9***	***n = 8***	***n = 9***	***n = 10***	***n = 9***	
Vitamin B2	1.1 ± 0.32[a]	0.33 ± 0.27 [A &]	0.33 ± 0.04 [a]	0.22 ± 0.04 [A]	0.32 ± 0.03 [a &]	0.24 ± 0.02 [A]	**P < 0.01[#]**
Vitamin B12	0.74 ± 0.13[a]	0.40± 0.07 [A]	0.25 ± 0.10 [b]	0.35 ± 0.74 [A]	0.18 ± 0.04 [b]	0.16 ± 0.02 [A]	P_{PCE} = 0.8; **P_{Chow} < 0.001**; $P_{PCE\ x\ chow}$ = 0.09
choline	1.0 ± 0.29[a]	0.62 ± 0.27[A]	0.28 ± 0.03[b]	0.29 ± 0.02[AB]	0.30 ± 0.01[b]	0.24 ± 0.01[B]	P_{PCE} = 0.1; P_{Chow} **< 0.001**; $P_{PCE\ x\ chow}$ = 0.2
Betaine	1.1 ± 0.20[a]	0.65 ± 0.08[A]	0.43 ± 0.08[b]	0.55 ± 0.04[A]	0.59 ± 0.02[ab]	0.51 ± 0.03[A]	**P < 0.05[#]**
DMG	1.0 ± 0.30[a]	0.57 ± 0.21[A]	0.3 ± 0.04[b]	0.29 ± 0.02[A]	0.33 ± 0.02[b]	0.27 ± 0.02[A]	P_{PCE} = 0.08; **P_{Chow} < 0.001**; $P_{PCE\ x\ chow}$ = 0.4
Methionine	0.87 ± 0.22	0.46 ± 0.07	0.45 ± 0.08	0.42 ± 0.04	0.53 ± 0.03	0.54 ± 0.06	P_{PCE} = 0.2; P_{Chow} = 0.12; $P_{PCE\ x\ chow}$ = 0.2
Hcy	1.3 ± 0.21[a]	0.54 ± 0.16[A]	0.65 ± 0.14[a]	0.43 ± 0.05[A]	1.59 ± 0.27[a]	1.51 ± 0.24[B]	**P_{PCE} = 0.02**; **P_{Chow} < 0.001**; $P_{PCE\ x\ chow}$ = 0.11
Vitamin B6	0.55 ± 0.19	0.19 ± 0.03	0.26 ± 0.03	0.28 ± 0.05	0.33 ± 0.04	0.28 ± 0.03	P_{PCE} = 0.11; P_{Chow} = 0.7; $P_{PCE\ x\ chow}$ = 0.3
SAM:SAH [^]	1.0 ± 0.22	1.25 ± 0.23	2.27 ± 0.60	1.45 ± 0.42	1.43 ± 0.40	1.40 ± 0.42	P_{PCE} = 0.4; P_{Chow} =0.5; $P_{PCE\ x\ chow}$ = 0.5

Std = standard chow (1.6 g choline/kg chow); Int = intermediate chow (2.6 g choline/kg chow); Supp = supplemented chow (7.2 g choline/kg chow); Hcy = homocysteine; SAM:SAH = s-adenosylmethionine: s-adenosylhomocysteine ratio.

[#] Kruskal-Wallis test for non-parametric data. Chow effects between Con groups shown by lower-case letters, and between PCE groups by upper-case letters.

[^] SAM and SAH could not be detected in all samples. For males: Std-Con *n*=4; Std-PCE *n* = 7; Int–con *n* = 5; Int-PCE *n* = 7; Supp-Con *n* = 8; Supp-PCE *n* = 9. For females: Std-Con *n*=6; Std-PCE *n* = 4; Int–Con *n* = 6; Int-PCE *n* = 7; Supp-Con *n* = 6; Supp-PCE *n* = 6.

[&] peak not detected for one sample.

Table S3: The effects of periconceptional ethanol and choline supplementation on components of the one-carbon metabolism (1CM) pathway in the fetal brain. During the periconceptional period**,** dams received a control liquid diet (Con) or a liquid diet containing 12.5% v/v ethanol (PCE). Chow levels varied from 1.6 g to 7.2 g choline/kg chow. Molecules measured by mass-spectrometry. Data expressed as fold-change relative to the average of the male Std-Con group (mean ± SEM).

**1CM Molecules**	**Int–Con**	**Std-PCE**	**Int–Con**	**Int-PCE**	**Supp-Con**	**Supp-PCE**	***P* Values**
Male	***n = 8***	***n = 10***	***n = 4***	***n = 5***	***n = 6***	***n = 5***	
Folic acid	1.0 ± 0.27 [&]	1.21 ± 0.29 [&&]	1.14 ± 0.11	0.66 ± 0.24 [&]	0.75 ± 0.23 [&]	1.35 ± 0.16	P = 0.4 [#]
Vitamin B2	1.0 ± 0.06[a]	1.16 ± 0.05[A]	0.86 ± 0.09[a]	0.70 ± 0.02[B]	0.95 ± 0.11[a]	0.94 ± 0.08[AB]	P_{PCE} = 0.9; **P_{Chow} < 0.001**; $P_{PCE\ x\ chow}$ = 0.9
Vitamin B12	1.0 ± 0.22	1.20 ± 0.15	1.32 ± 0.29	1.32 ± 0.29	1.45 ± 0.13	0.86 ± 0.15	P_{PCE} = 0.4; P_{Chow} =0.1; $P_{PCE\ x\ chow}$ = 0.95
choline	1.0 ± 0.03	1.1 ± 0.06	0.92 ± 0.10	0.87 ± 0.09	1.10 ± 0.08 [&]	1.06 ± 0.09	P_{PCE} = 0.9; **P_{Chow} < 0.05**; $P_{PCE\ x\ chow}$ = 0.4
Betaine	1.0 ± 0.03	1.19 ± 0.05	0.91 ± 0.08	1.12 ± 0.19	1.02 ± 0.05	1.14 ± 0.10	**P_{PCE} = 0.03**; P_{Chow} =0.19; $P_{PCE\ x\ chow}$ = 0.9
DMG	1.0 ± 0.03	1.10 ± 0.05	0.93 ± 0.13 [$]	0.86 ± 0.09	1.10 ± 0.08	1.03 ± 0.08	P_{PCE} = 0.99; P_{Chow} =0.12; $P_{PCE\ x\ chow}$ = 0.4
Methionine	1.0 ± 0.06[a]	0.89 ± 0.05[A]	0.75 ± 0.04[a]	1.02 ± 0.11[A]	1.37 ± 0015[b]	1.47 ± 0.23[A]	**P = < 0.01** [#]
Vitamin B6	1.0 ± 0.30[a]	0.95 ± 0.14[A]	0.84 ± 0.29[b]	0.60 ± 0.12[A]	0.75 ± 0.17[b]	0.85 ± 0.40[A]	P_{PCE} = 0.66; P_{Chow} =0.36; $P_{PCE\ x\ chow}$ = 0.8
Female	***n = 8***	***n = 9***	***n = 4***	***n = 5***	***n = 6***	***n = 5***	
Folic acid	0.84 ± 0.16	0.89 ± 0.11	0.82 ± 0.13	0.97 ± 0.22	0.64 ± 0.16	1.03 ± 0.30	P_{PCE} = 0.26; P_{Chow} =0.70; $P_{PCE\ x\ chow}$ = 0.8
Vitamin B2	1.0 ± 0.07[a]	1.13 ± 0.04[A]	0.80 ± 0.09[a]	0.79 ± 0.05[B]	0.84 ± 0.06[a]	0.93 ± 0.07[AB]	P_{PCE} = 0.3; **P_{Chow} < 0.001**; $P_{PCE\ x\ chow}$ = 0.7
Vitamin B12	1.3 ± 0.32 [&]	1.06 ± 0.15	1.49 ± 0.08	1.49 ± 0.19	0.58 ± 0.11	1.02 ± 0.25	P_{PCE} = 0.8; P_{Chow} = 0.05; $P_{PCE\ x\ chow}$ = 0.4
choline	1.04 ± 0.09	0.95 ± 0.04	0.90 ± 0.11	0.94 ± 0.10	0.93 ± 0.07	0.92 ± 0.05	P_{PCE} = 0.8; P_{Chow} = 0.6; $P_{PCE\ x\ chow}$ = 0.7
Betaine	1.06 ± 0.12	1.23 ± 0.11	0.88 ± 0.10 [&]	1.07 ± 0.08	1.02 ± 0.05	1.04 ± 0.05	P_{PCE} = 0.12; P_{Chow} =0.4; $P_{PCE\ x\ chow}$ = 0.6
DMG	1.0 ± 0.09	0.94 ± 0.03	0.81 ± 0.10 [&]	0.92 ± 0.10	0.91 ± 0.07	0.90 ± 0.05	P_{PCE} = 0.92; P_{Chow} =0.34; $P_{PCE\ x\ chow}$ = 0.5
Methionine	1.10 ± 0.12	1.28 ± 0.21	0.98 ± 0.17	0.97 ± 0.12	1.16 ± 0.11	1.08 ± 0.15	P_{PCE} = 0.94; P_{Chow} =0.46; $P_{PCE\ x\ chow}$ = 0.7
Vitamin B6	0.84 ± 0.36	0.82 ± 0.20	0.99 ± 0.29	1.07 ± 0.31	0.75 ± 0.21	0.82 ± 0.20	P_{PCE} = 0.49; P_{Chow} =0.55; $P_{PCE\ x\ chow}$ = 0.98

Std = standard chow (1.6 g choline/kg chow); Int = intermediate chow (2.6 g choline/kg chow); Supp = supplemented chow (7.2 g choline/kg chow); Hcy = homocysteine; SAM:SAH = s-adenosylmethionine: s-adenosylhomocysteine ratio.

[#] Kruskal-Wallis test across all 6 groups for non-parametric data. Chow effects between Con groups shown by lower-case letters, and between PCE groups by upper-case letters.

[&] Peak not detected for 1 sample; [&&] peak not detected for 2 samples.
